# A protocol for visualization of murine *in situ* neurovascular interfaces

**DOI:** 10.1016/j.xpro.2023.102367

**Published:** 2023-06-19

**Authors:** Danielle D. Dang, Vikram Chandrashekhar, Vibhu Chandrashekhar, Nagela Ghabdanzanluqui, Russell H. Knutsen, Matthew A. Nazari, Likitha Nimmagadda, Danielle R. Donahue, Dorian B. McGavern, Beth A. Kozel, John D. Heiss, Karel Pacak, Zhengping Zhuang, Jared S. Rosenblum

**Affiliations:** 1 Neuro-Oncology Branch, National Cancer Institute, National Institutes of Health, Bethesda, MD 20892, USA; 2 Surgical Neurology Branch, National Institute of Neurological Disorders and Stroke, National Institutes of Health, Bethesda, MD 20892, USA; 3 Neurosimplicity LLC, Shrewsbury, NJ 07702, USA; 4 Laboratory of Vascular and Matrix Genetics, National Heart Lung and Blood Institute, National Institutes of Health, Bethesda, MD 20892, USA; 5 Mouse Imaging Facility, National Institute of Neurological Disorders and Stroke, National Institutes of Health, Bethesda, MD 20892, USA; 6 Viral Immunology and Intravital Imaging Section, National Institute of Neurological Disorders, National Institutes of Health, Bethesda, MD 20892, USA; 7 *Eunice Kennedy Shriver* National Institute of Child Health, Bethesda, MD 20892, USA

**Keywords:** Model Organisms, Neuroscience, Systems biology

## Abstract

Mapping cranial vasculature and adjacent neurovascular interfaces in their entirety will enhance our understanding of central nervous system function in any physiologic state. We present a workflow to visualize *in situ* murine vasculature and surrounding cranial structures using terminal polymer casting of vessels, iterative sample processing and image acquisition, and automated image registration and processing. While this method does not obtain dynamic imaging due to mouse sacrifice, these studies can be performed before sacrifice and processed with other acquired images.

For complete details on the use and execution of this protocol, please refer to Rosenblum et al.^1^

## Highlights

• Vascular casting and micro-CT allow visualization of vascular anatomy
• Non-invasive visualization of *in situ* cranial vasculature and surrounding anatomy
• Non-destructive iterative sample processing allows repeated image acquisition
• Tools for automated image processing, feature extraction, and visualization

## Abstract (editor highlights)

Publisher’s note: Undertaking any experimental protocol requires adherence to local institutional guidelines for laboratory safety and ethics.

## Graphical abstract

**Figure:**
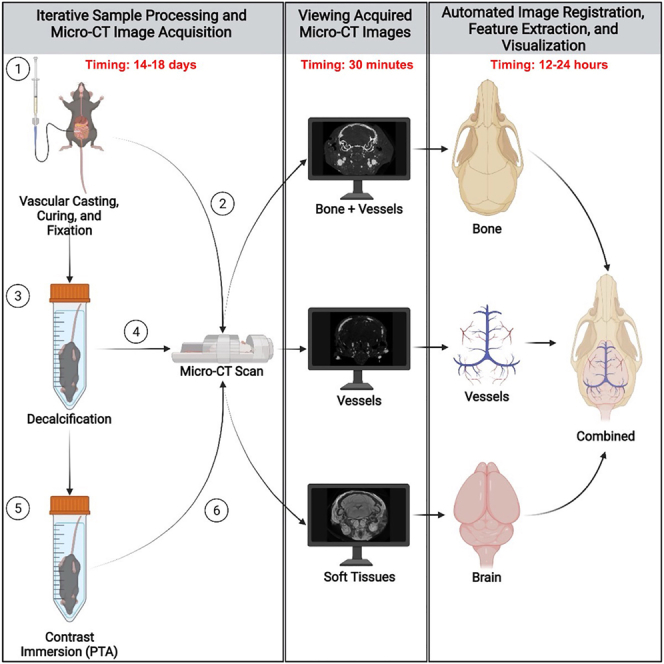

## Teaser

Mapping cranial vasculature and adjacent neurovascular interfaces in their entirety will enhance our understanding of central nervous system function in any physiologic state.^2^ We present a workflow to visualize *in situ* murine vasculature and surrounding cranial structures using terminal polymer casting of vessels, iterative sample processing and image acquisition, and automated image registration and processing. While this method does not obtain dynamic imaging due to mouse sacrifice, these studies can be performed before sacrifice and processed with other acquired images.^3^

## Before you begin

**Timing: 1–2 h**

1. Prepare the murine model of interest for euthanasia, terminal vascular casting, and fixation (Figure 1).

**Figure 1:**
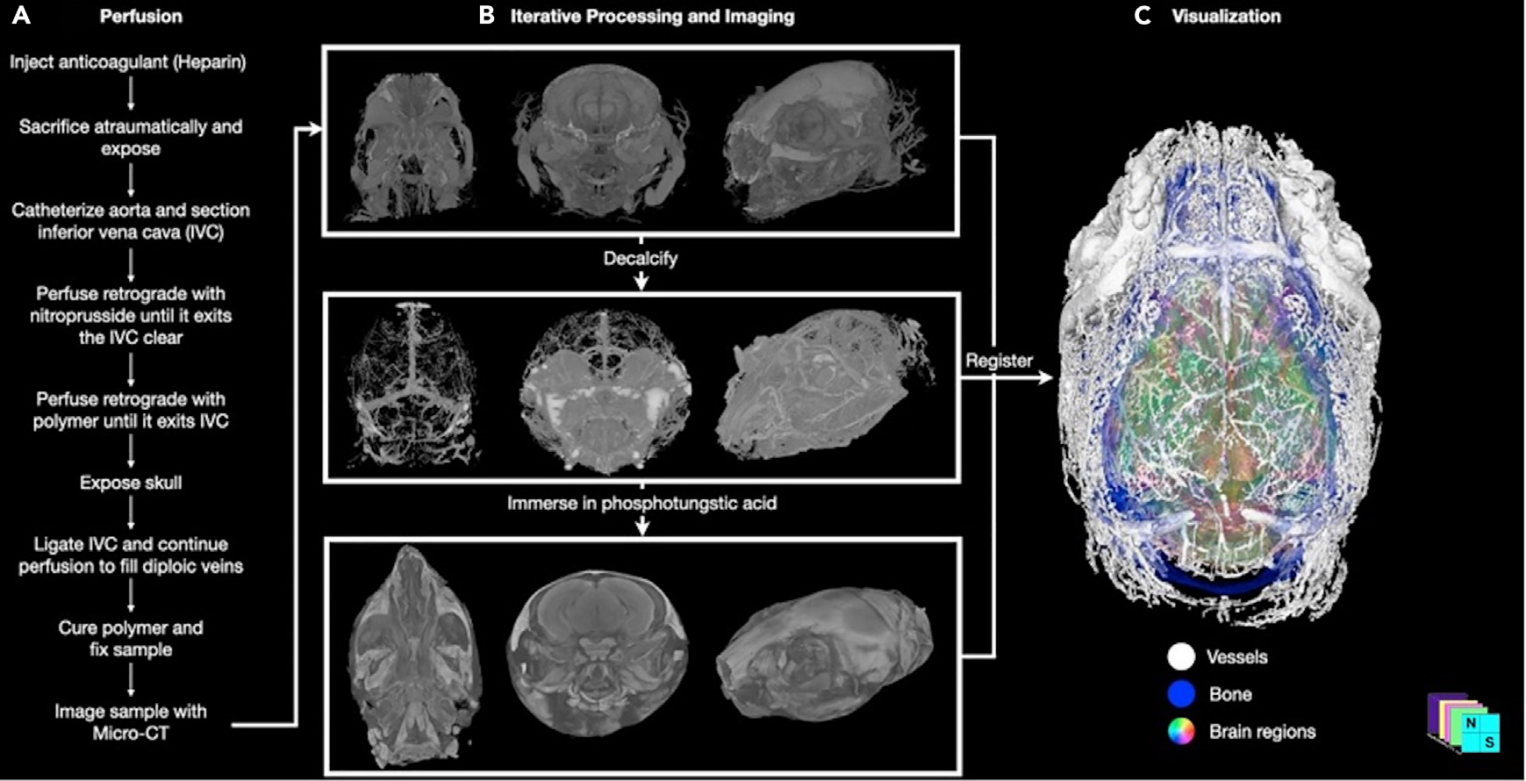
Overview of the major step-by-step methodology for non-invasive *in situ* visualization of murine cranial vasculature (A) Steps for cranial vascular casting perfusion are shown. (B) Iterative sample processing and imaging. The same representative wild-type mouse sample is shown. The top row shows a 3D reconstruction of a whole head micro-CT with Microfil polymer-casted cranial vasculature and bone. Micro-CT after decalcification shown in the middle row highlights the vasculature in isolation. Immersion in phosphotungstic acid (PTA) makes the tissues radio-opaque, as shown in the bottom row. (C) 3D visualization of all datasets deformably registered to the same space displaying the segmented vessels (white), bone (blue), and brain regions derived from the Allen Reference Atlas Common Coordinate Framework version 3 (ARA CCF v3) (multi-color). *Published with permission from Rosenblum* et al.*, 2022.*
2. Set up the dissection and perfusion station in accordance with the key resources table.
3. Prepare solutions for iterative sampling. Prepare the polymer mixture immediately before perfusion to avoid curing.
4. Confirm functionality of the micro-computed tomography (micro-CT) by turning on the machine and confirming optimal settings.

### Institutional permissions

Experiments on mice received appropriate institutional approvals and were performed in accordance with institutional guidelines.

Prior to executing the protocol presented herein, investigators must also acquire relevant permissions from their institution and follow IACUC guidelines and regulations.

### Perfusion preparation

**Timing: 5 min**

1. Set-up the perfusion station with the following materials:
  a. Surgical board.
  b. Surgical tape.
  c. #10 or #15 blade scalpel(s).
  d. Operating microscope.
  e. CeramaCut scissors 9 cm (cm).
  f. Ceramic coated curved forceps.
  g. Dumont mini forceps.
  h. Octagon forceps straight teeth.
  i. Straight spring scissors.
  j. Hemostat.
  k. 7-0 silk sutures (4).
  l. 5 mL (mL) syringe.
  m. 10 mL syringe.
  n. 30-gauge (G) needle.
  o. Catheter - 15 cm polyethylene-10 (PE-10) tubing attached to 30G needle.
  p. Tape.
2. Load a 10 mL syringe with 10^−4^ M sodium nitroprusside in a phosphate-buffered saline (PBS), attach the 30G needle/PE-10 tubing, purge all air bubbles, and set aside at 20°C–22°C.
3. Set aside reagents to be used for the Microfil® silicate polymer mixture at 20°C–22°C. Materials and equipment: Table 2.

***Note:*** While any type of dissector tool can be utilized, we recommend the use of ceramic-coated tools when manipulating blood vessels to decrease inadvertent adherence and vessel injury.

***Note:*** Sodium nitroprusside is used to maximally dilate the blood vessels and to flush blood efficiently from the vascular system.

### Curing and fixation preparation

**Timing: 5 min**

4. Set up the container to be used for Microfil® curing and sample fixation and set aside.
  a. Place dry ice at the bottom of a Styrofoam container.
  b. Cut a piece of cardboard to fit the shape of the container.
  c. Place the cardboard barrier on top of the dry ice.
5. Set aside fixative of choice (i.e., 4% paraformaldehyde, 10% formalin) and PBS.

### Mouse euthanasia

**Timing: 5 min**

6. Weigh the mouse to be studied.
7. Inject heparin (1 unit/g) into the intraperitoneal space of the mouse and wait 2 min.
8. Sacrifice the mouse via carbon dioxide narcosis.
9. When the mouse has stopped moving and there is no visible respiration, verify successful euthanasia by conducting a paw pinch maneuver; there should be no reflexive withdrawal. If the reflex is present, repeat steps 3–4 above.

***Note:*** All mice should be housed in the following conditions: a dedicated, pathogen-free animal facility with a 12/12-h light/dark cycle and food and water provided at libitum or in accordance with the mouse protocol in use.

***Note:*** Heparin is used to anticoagulate the blood and allow for efficient clearing prior to perfusion and vascular casting.

**CRITICAL:** For best results, perfusion and vascular casting should promptly follow euthanasia. Mice preservation is not required during this time interval.

## Key resources table

**Table:** 

REAGENT or RESOURCE	SOURCE	IDENTIFIER
**Chemicals, peptides, and recombinant proteins**		

CO_2_ tank	Robert’s Oxygen Co	N/A
Ethanol	Pharmco	Cat#111000200
Formalin	Sigma-Aldrich	Cat#HT501128
Heparin (1000 USP Units/mL)	Hospira NDC	Cat#0409-2720-01
Hydrochloric acid	Sigma-Aldrich	Cat#V800203
Microfil® (polymer compound)	Flow Tech, Inc.	Cat#KitB-MV-122
Paraformaldehyde	Sigma-Aldrich	Cat#P6148-500G
Phosphate-buffered saline	Bio-Rad	Cat#161-0780
Phosphotungstic acid	Sigma-Aldrich	Cat#P4006-25G
Sodium azide	Sigma-Aldrich	Cat#26628-22-8
Sodium nitroprusside	Sigma-Aldrich	Cat#71778-25G

**Experimental models: Organisms/strains**		

Murine model to be studied	N/A	N/A

**Software and algorithms**		

Neurosimplicity Imaging Suite	Neurosimplicity, LLC	www.neurosimplicity.com

**Other**		

1 mL syringe	Becton Dickinson	Cat#309659
10 mL syringe	Becton Dickinson	Cat#303134
30G needle	Becton Dickinson	Cat#305106
50 mL conical tubes	Corning	Cat#352098
7-0 silk suture	Teleflex	Cat#103-S
#10 scalpel blade	Fine Science tools	Cat#10010-00
CeramaCut scissors 9 cm	Fine Science tools	Cat#14958-09
Ceramic coated curved forceps	Fine Science tools	Cat#11272-50
Dual syringe pump	Cole Parmer	Cat#EW-74900-10
Dumont mini forceps	Fine Science tools	Cat#11200-14
Gauze	Covidien	Cat#441215
Halsted-Mosquito hemostat, straight	Fine Science tools	Cat#13008-12
Induction chamber	–	–
Kimwipe	Fisher	Cat#06-666
Labeling tape	Fisher	Cat#15966
Magnetic base	Kanetec	–
Micro-CT system	Bruker	–
Micromanipulator	Stoelting	Cat#56131
Octagon forceps straight teeth	Fine Science tools	Cat#15000-08
Parafilm	Bemis company, Inc.	Cat#PM999
PE-10 tubing	Instech	Cat#BTPE-10
Ring stand	Fisher	Cat#S13747
Steel plate (16 × 16 in. area, 1/16 thickness)	–	–
Straight spring scissors	Fine Science tools	Cat#13013-14
Surgical board	Fisher	Cat#12-587-20
Zeiss Stemi-508 Dissection Scope	Zeiss	–

## Materials and equipment

**Table:** 

Microfil silicate polymer mixture		
Reagent	Final concentration	Amount
Microfil ®	20%	200
Diluent	70%	700
Polymerizing agent	10%	100
**Total**	**100%**	**1 mL**

***Note:*** Storage is not necessary as this solution is made on each use.

***Note:*** Alterations to above percentages can be made depending on the goal of polymer casting.^2^^,^^3^^,^^4^^,^^5^ Increasing concentrations of Microfil® will isolate the arterial vasculature while lower concentrations will cross capillaries and allow for visualization of both arterial and venous circulation. The above 2 to 7 to 1 ratio of Microfil® to diluent to polymerizing agent is best suited for arterial-venous transit.^3^

***Alternatives:*** Any radio-dense polymer or contrast agent may be used to cast the vessels though concentrations will have to be adjusted.^6^ An example of another polymer that may be used is MicroAngiofil (MediLumine, Inc, Montreal, Canada).

### Other solutions

• 10% Hydrochloric acid (HCl): Prepare a 10% v/v dilution from stock 35%–37% HCl with deionized water.
• 1× PBS: Prepare a 10-fold dilution from stock 10× PBS with deionized water.
• 70% Ethanol: Prepare 70% v/v solution of ethanol 200 proof in de-ionized water.
• 50% Ethanol: Either prepare 50% v/v solution from stock ethanol 200 proof or dilute previously made 70% ethanol solution.
• 30% Ethanol: Either prepare 30% v/v solution from stock ethanol 200 proof or dilute previously made 70% or 50% ethanol solution.
• Sodium Nitroprusside in PBS: Prepare a 10^−4^ M sodium nitroprusside in 1× PBS solution as prepared above.
• Phosphotungstic acid (PTA) in Ethanol or PBS: Dilute PTA hydrate in 70% ethanol (made from stock ethanol 200 proof) or in 1× PBS to desired concentration (0.3%–0.7% recommended).

***Note:*** All solutions above should be made on each use and therefore should not require long-term storage. If storage is necessary, solutions without the sample can be stored for up to six months at 20°C–22°C.

**CRITICAL:** All acidic solutions above should be handled with standard laboratory precautions including gloves and protective eyewear to avoid exposure to mucous membranes.

***Note:*** For any solution in which one sample is to be immersed, we recommend at least 50 mL or an amount in which the sample will be adequately submerged.

***Note:*** PTA solution can be made in organic or aqueous solvents such as ethanol or PBS. Both options are provided in the table. Ethanol may lead to tissue shrinkage artifacts apparent on imaging or subsequent histology.^7^^,^^8^^,^^9^

***Note:*** To mitigate microbial growth, samples should be kept at 4°C during processing. In addition, 0.02% sodium azide should be added to all solutions while samples are processed.

***Alternatives:*** Herein, decalcification is performed with 10% HCl; however, this may cause tissue degradation artifacts that may be apparent in subsequent histology. This can be mitigated by using a lower percent HCl solution (e.g., 5%) or by using other decalcifying solutions such as ethylenediaminetetraacetic acid (EDTA) at a range of concentrations (e.g., 20%–25%). Both methods may require more time to achieve decalcification.

### Micro-CT settings

• Settings must adhere to the following principles for optimal radiographic transmission:
  • When each material within a given field of view has its own unique radiodensity without saturation, which is defined as the maximum absorption or transmission, X-ray transmission has been optimized as shown in the table below.
  • Maximization of resolution for smaller features in the image, which is accomplished by having a minimum of 2–4 pixels/voxels for the smallest feature of interest.
  • Application of appropriate filters relative to accelerating voltage (kV) and sample radiodensity which have a linear relationship with each other and with the thickness of the filter required. We use an aluminum filter which is thinner and provides less filtration.
• Optimize x-ray settings, exposure time, and power settings to allow sufficient transmission through the sample and allow resolution of the Microfil, bone, and PTA per the above criteria (Table 1).

**Table 1:** Representative micro-CT settings used for samples presented in the study

Representative micro-CT settings used for samples presented in the study		
Settings	Sample type	
	Microfil® Perfused (with or without decalcification)	PTA
Micro-CT Instrument	SkyScan 1172	Skyscan 1272
X-ray source	5μm focal spot, energy range 20–100 kV	
Power	65kV/153 μA	100kV/100 μA
Filter	Al 0.5 mm	Cu 0.11 mm
Resolution (pixel size)	13.53 μm	6.999978 μm
Camera-to-Source Distance	345.233 mm	175.06189 mm
Object-to-Source Distance	259.500 mm	83.59000 mm
Pixel Binning	2 × 2	2 × 2
Scan Orbit	360 degrees	360 degrees
Rotation Step	0.4 degrees	0.175 degrees
Averaging	6 frames	10 frames
Exposure Time	1600 ms/frame	1275 ms/frames

***Note:*** Given the structure and shielding of the micro-CT machine, radiation precautions such as a lead apron or a badge are generally not required during use, but the user should check with their institutional safety board.

***Alternatives:*** The scans described herein were performed using a SkyScan 1172 and 1272 Micro-CT scanners. Any micro-CT machine can accommodate the workflow as long as the radiographic principles described above are followed. The instrument should have a variety of filter materials and thicknesses and be capable of multi-segment scanning.

### Neurosimplicity software specifications

• The Neurosimplicity Imaging Suite software utilizes automatic deformable registration, segmentation/feature extraction, and visualization to register the iteratively processed and acquired images to each other. The software further registers these samples to an atlas such as the Allen Reference Atlas Common Coordinate Framework version 3 (ARA CCF v3),^10^ extracts anatomic features of interest, and visualizes the resulting registered 2D raw data and 3D visualizations to produce a final result.
• The software automatically processes imaging using automated denoising and feature extraction applied to the raw 2D data to generate the 3D visualizations.
• Raw data files accepted for use by this software include but are not limited to bitmap, TIF, and DICOM.

### Other software specifications

• NRecon (Bruker MicroCT), which is used to reconstruct raw micro-CT data.
• DataViewer (Bruker MicroCT), which is used for 2D visualization of all 3 planar sections of reconstructed data. Note: this program can also be used to rotate the 2D data across all 3 planar directions, save rotated and/or smaller datasets, or make linear measurements.
• CTvox (Bruker MicroCT). Note: This is optional but can be used for 3D visualization of individual data in different light, material properties, opacities, and colors.

***Alternatives:*** There are no available alternative commercial or open-source programs to automatically perform deformable registration, feature extraction, and visualization on iteratively acquired imaging data of the same iteratively processed sample.

## Step-by-step method details

### Perfusion

**Timing: 45 min**

This section provides stepwise instructions to catheterize the abdominal vessels of the mouse and to terminally cast and visualize the intracranial vasculature with polymer mixture.

**Figure 2:**
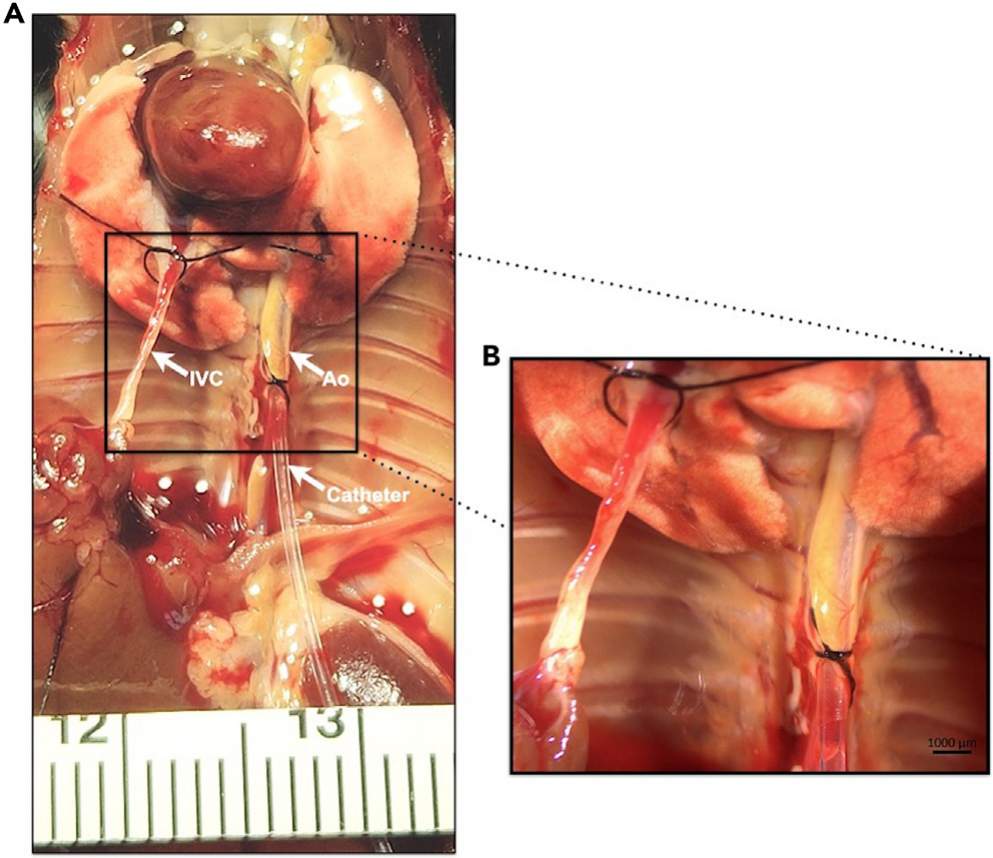
Catheterization for perfusion and casting (*A)* Gross photograph of the aorta (Ao) and inferior vena cava (IVC) following exposure and catheterization. The catheter is secured with suture. The IVC, which has not yet been incised to allow outflow of perfused solutions, has a loose suture placed, which is later secured when perfusion is complete. (*B)* Photograph of the Ao and IVC following catheterization under the dissecting microscope at 0.63× magnification. Scale bar = 1000 microns.
  g. If not previously prepared, fill a 10 mL syringe with 10^−4^ M sodium nitroprusside in a phosphate-buffered saline (PBS).
  h. Connect the 10 mL syringe to a 30G needle.
  i. Fasten the syringe with the needle to a catheter made of 15 cm of polyethylene-10 (PE-10) tubing.
  j. Expel air bubbles from the tubing connected to the syringe by flushing the catheter.
  k. Insert the PE-10 catheter into the incision previously made in the aorta.
2. Advance the catheter beyond the sutures and secure.
3. Perfuse the sample.
  a. Begin perfusing the vasculature with sodium nitroprusside solution to remove blood and achieve maximal vessel dilation. Troubleshooting 1.
    i. Continue until perfusate exiting the IVC is clear, approximately 10 min.
    ii. Leave the catheter in place once complete.
  b. Prepare a 5 mL mixture of Microfil® silicate polymer as follows: 2 to 7 to 1 ratio of Microfil® to diluent to polymerizing agent.
  c. Remove the plunger from a 5 mL syringe and fill with the above mixture. Replace the plunger, invert the syringe, and purge the syringe of air.
  d. While being careful not to introduce air bubbles, disconnect the 10 mL syringe from the 30G needle and replace with the 5cc syringe filled with Microfil®.
  e. Slowly perfuse until Microfil® can be seen exiting the IVC, approximately 15 min from the start of perfusion. Troubleshooting 2 and 3.
    i. Ligate the IVC by securing the suture circumferentially to stop polymer outflow.
  f. Position the mouse prone.
    i. Tent the scalp and incise midline, exposing the skull from the snout to the neck to the level of the galea aponeurotica, which has many veins.

***Note:*** Preservation of the galea prevents small leaks from the venous system and still allows diploic veins and dural venous sinuses to be appreciated through the thin murine skull (Figure 3).

***Note:*** Blood within diploic veins, or venous structures present between the boney layers of the skull, can be seen as it is replaced with polymer. Continue to inject Microfil® until all visible vascular structures are filled with polymer.

***Note:*** Transgression of the galea aponeurotica will afford a better view of the diploic veins but may facilitate small vascular leaks which will decrease perfusion pressure and possibly lead to incomplete vessel filling. To instead avoid disruption of this tissue, and therefore small vessel leaks, incise above the galea aponeurotica by maintaining dissection within the loose areolar plane containing the fatty scalp tissue only. This step is dependent on operator ability. We recommend keeping the galea intact, however, if it is divided, it will not significantly impact terminal vessel casting.

1. Positioning and exposure of the vasculature to be catheterized.
  a. Position the mouse supine on a surgical board and secure all four limbs with tape.
  b. Under microscopic visualization, perform a midline abdominal incision with a #10 or #15 scalpel extending to the xyphoid process.
  c. Using a hemostat, secure the xyphoid process.
    ***Optional:*** If access to the abdominal aorta and inferior vena cava (IVC) is limited and greater exposure is therefore needed, incise the diaphragm and rib cage bilaterally inferior to the internal thoracic arteries. The anterior chest wall can then be elevated to expose the thoracic cavity.
  d. Identify and secure the IVC, close to the diaphragm, with a 7-0 silk suture. Place an additional loose suture around the IVC, proximal to the heart.
  e. Make an incision in one wall of the IVC in between the two placed sutures.
  f. Similarly, place two loose sutures around either the exposed abdominal or thoracic aorta and make a small incision distal to those sutures (Figure 2).

***Optional:*** A syringe pump may be used as an alternative to manually driving the Microfil®.

**Figure 3:**
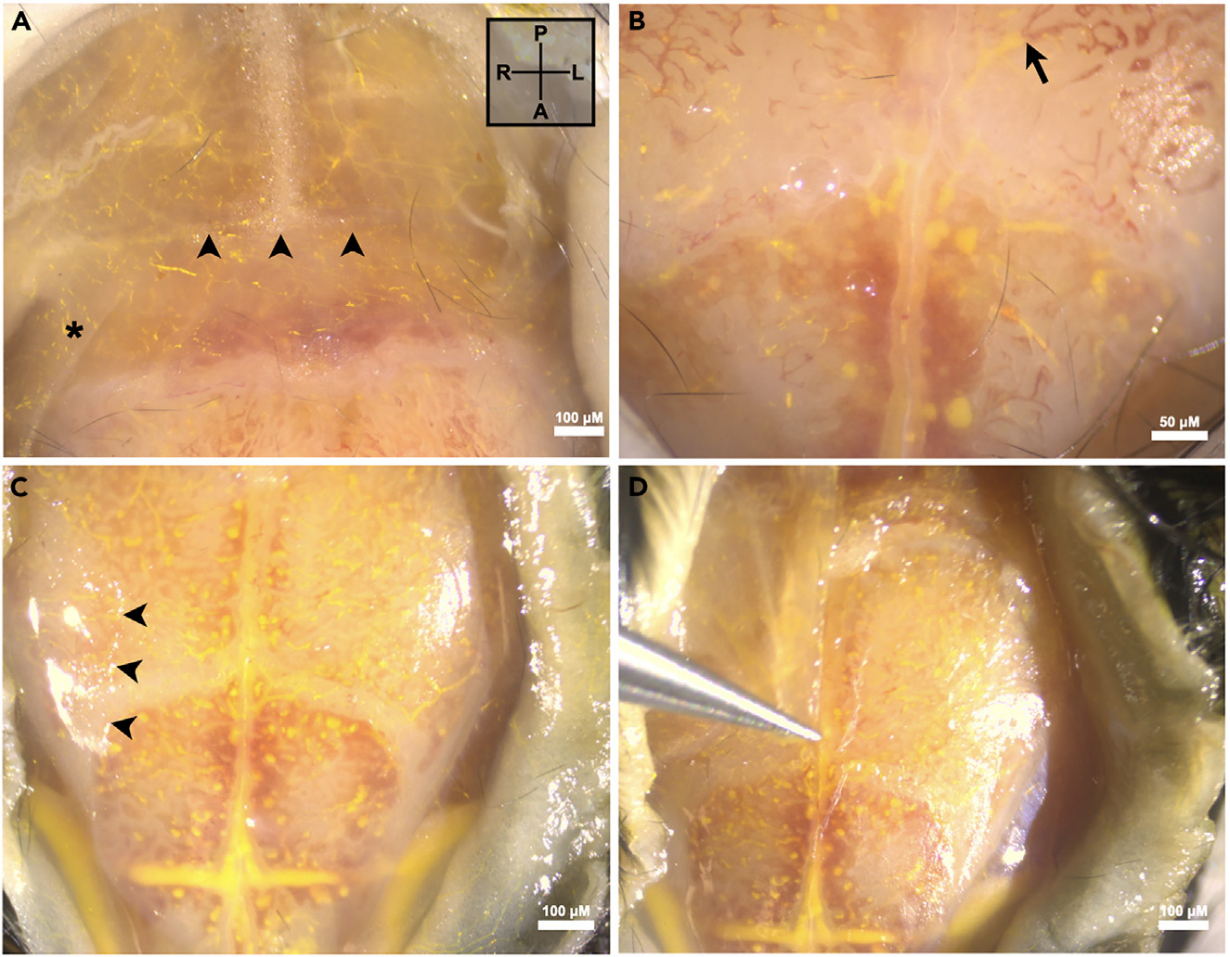
Discerning the galea aponeurotica (A) Anatomic view of the top of the posterior aspect of the murine skull centered over the craniocervical junction where the galea aponeurotica inserts into the occipitocervical fascia (arrowheads). A reflection of the galea denotes its delicate transparent appearance as it stretches anteriorly over the pericranium (asterisk). (B) Partial polymer filling of diploic veins (arrow) in a galea-preserved specimen. (C) Division of the galea after complete polymer casting. The galea (arrowheads) was lateralized on both sides for direct comparison of vessel casting visualization with and without this additional layer. With the galea preserved, visualization is decreased due to the additional layer of tissue and increased light reflection, however, small vessel leaks are minimized and hydration of the pericranium is concomitantly preserved. (D) The galea is elevated from the murine skull and grasped with forceps to demonstrate its tensile strength. *Abbreviations: A-Anterior; L-Left; R-Right; P-Posterior.*

### Microfil® curing and sample fixation

**Timing: 25–37 h**

This section provides stepwise instructions to cure the vascular casting solution and to preserve the sample in preparation for iterative processing and imaging.

4. Allow the Microfil® to cure for approximately 60 min. Troubleshooting 4.

***Note:*** The mouse (sample) may be cured using ice, dry ice, or at 4°C to prevent tissue decay to suit the needs of downstream applications. Troubleshooting 5.

***Note:*** If utilizing dry ice for storage, consider placement of samples on top of a cardboard barrier to prevent direct contact of the sample with dry ice and therefore rapid ice formation. Rapid freezing may create histologic artifacts.

5. Fix the sample in a volume sufficient to immerse the entire mouse for 24–36 h at 20°C–22°C. or at 4°C using a fixative appropriate for downstream applications.

***Note:*** When immunohistochemistry is anticipated, consider fixation in 4% paraformaldehyde using the same parameters.

***Note:*** When only cytomorphologic assessment is anticipated, consider 24-h fixation in 10% formalin.

***Note:*** Antigen retrieval may be performed if needed when immunohistochemistry is performed on a sample that has been fixed in formalin.

6. Transfer samples into PBS, add 0.02% sodium azide, and store in 4°C until imaging.

***Note:*** Specimen storage duration is not restricted as long as the fixative is appropriate and microbial growth can be prevented. Imaging quality does not decrease with the duration of storage, and samples can be reimaged at later dates if desired.

### Micro-CT set-up

**Timing: 30–45 min**

This section provides stepwise instructions to set-up the micro-CT and optimize its parameters for use at every stage of sample processing.

7. Turn on the micro-CT and begin warming/aging of the X-ray source per manufacturer instructions.
8. Prepare and place the sample in an appropriate specimen holder, noting the following:
  a. The orientation of the sample should be as symmetrical as possible and perpendicular to the path of the X-ray beam to maintain similar X-ray penetration depth throughout the scan rotation.
  b. The holder must 1.) be rigid and as radiolucent as possible, 2.) prevent sample movement and any changes in hydration status and 3.) will ideally be no wider and/or longer than necessary to allow the sample and air on each of its sides to be visible within the field of view (FOV) along the path of the X-ray beam. Note: Parafilm can be used instead of a tube with a top.
9. Place sample in the scanner and determine appropriate settings based of the X-ray absorption through the sample while looking at its radiograph/scout and adjusting as needed.
  a. Pixel Size and Pixel Matrix: determine based on sample size, scanner capabilities and study needs
  b. Filter Setting, X-ray Voltage (kV), Power (μA), Exposure Time (ms): air should have the lowest absorbance, while the rest of the sample should have an increase in absorption through each of its different radio density characters throughout the sample (Figure 4). The most radio dense areas will absorb while still allowing sufficient beam penetration past these areas and through the entirety of the sample.

**Figure 4:**
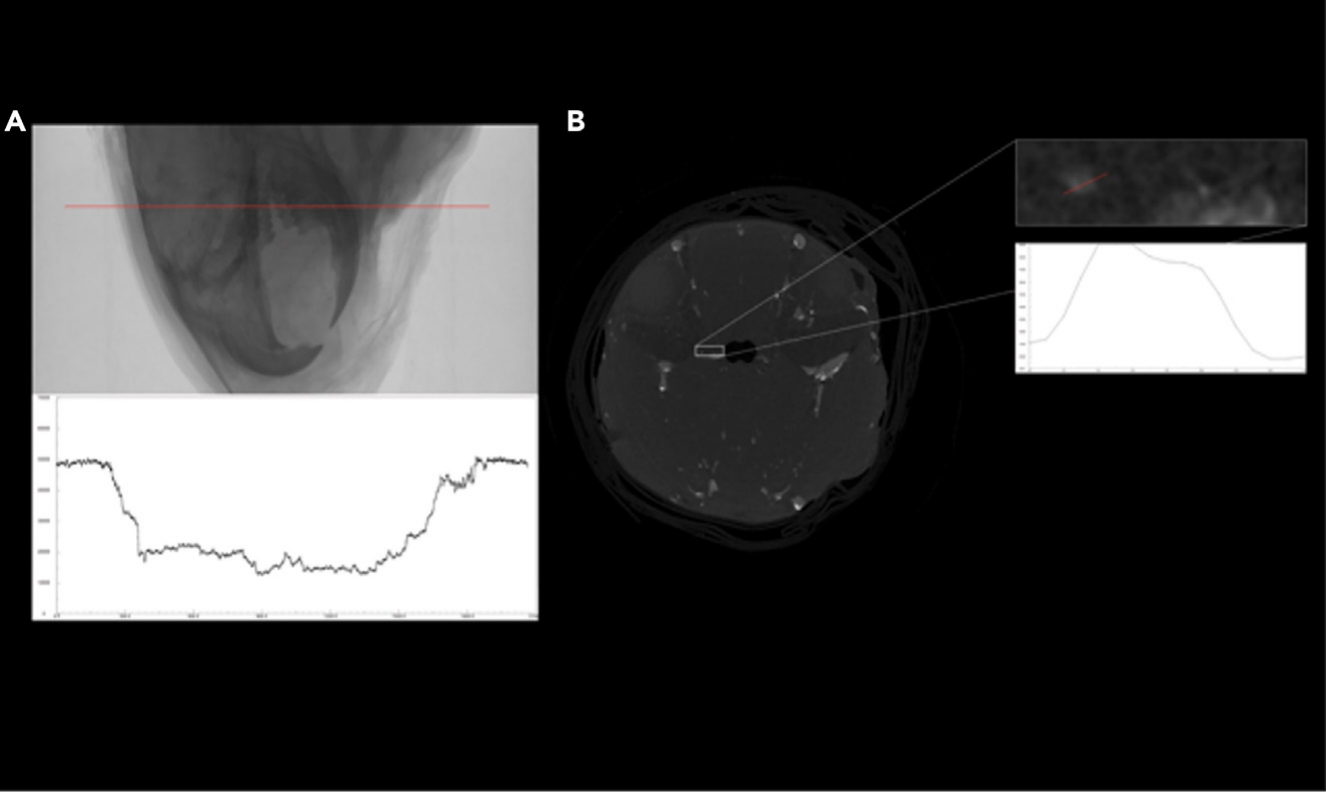
Optimization of micro-CT parameters for iterative sample processing and imaging (A) X-Ray projection image of murine head after vascular polymer casting and fixation but before decalcification and PTA immersion. The plot below shows the intensity profile of the above image at the pixels along the red line shown. (B) A single coronal slice reconstructed from the micro-CT of the same sample after decalcification. The inset and plot on the right show the intensity profile of a small vessel indicated by the red line. Related to STAR Methods.
10. Remove the sample and all materials from the FOV, allowing only air to remain and obtain calibration images per manufacturer instructions at the determined scan settings. This may include a bright image (X-ray on) and dark image (X-ray off).
11. Place sample within the FOV.
12. Enter additional scan parameters as determined by your study needs:
  a. Scan Name.
  b. Rotation Step.
  c. Averaging.
  d. Random Movement/Dead Pixel Compensation.
  e. Total Rotation (180º vs. 360º).
13. Turn on the X-ray source and allow the sample to reach a stable state.
14. Begin scanning.

### Iterative processing and imaging

**Timing: 12–16 days**

**Timing: ∼12–24 h for step 15**

**Timing ∼30–45 min for step 16**

**Timing ∼3–5 days for step 17**

**Timing ∼ 8–10 days for step 18**

This section provides stepwise instructions for iterative sample processing and imaging of brain, bone, and vessels within the sample.

***Note:*** If a scan with calcified bone and polymer-perfused vasculature is desired, continue to the next step below.

***Note:*** If only the polymer-perfused vasculature without bone is desired, proceed directly to step 8.

***Note:*** To add soft tissue visualization, continue to step 9.

15. Perform micro-CT on the sample.
  a. To prevent movement of the sample during image acquisition, secure the sample in an appropriately fitted holder that fits within the scanner FOV. Consider wrapping the sample in parafilm and securing it in a trimmed 50 mL conical tube.
  b. Determine the optimal scanning parameters for your sample. Acquire bright field.
    ***Note:*** Time to scan the sample will largely depend on the number of FOV sections required to capture the entire sample. A partial volume scan will decrease the scan time, but the sample must be centered; a micro-positioner attachment can help.
  c. Check your scan settings, including the file names and output location.
  d. Start the scan. Troubleshooting 6.
  e. When the scan is complete, remove the sample, and return it to PBS.
16. Reconstruction of raw data and preliminary visualization.
  a. Open images in NRecon.
  b. Use the densest material, usually the enamel, to adjust the dynamic range by setting the upper limit.
    i. Apply ring artifact reduction and beam hardening correction as needed.
  c. Fine tune the alignment of each section of the scan.
  d. Start the reconstruction.
    ***Note:*** Maintain the same parameters between the samples, including the beam hardening correction, dynamic range, and smoothing parameters. Reconstruction time is largely dependent on the number of sections contained in the scan, image resolution, and computational power.
    ***Optional:*** If desired, rotate the reconstructed images in DataViewer® 2D/3D micro-CT Slice Visualization (Bruker Software, Microphotonics, Allentown, PA) and export the newly rotated reconstruction.
  e. SkyScan CTVox software (Microphotonics, Allentown, PA) or any other software capable of viewing the image files may be used to generate and view the volume rendered 3D images of each acquired image.
    i. Adjust parameters in the thresholding window to accentuate the Microfil® and remove soft tissue.
      ***Note:*** Preset transfer functions may be provided with the software package.
      ***Note:*** Microfil® and bone tend to have similar attenuation and the skull may not be completely removed by adjusting thresholding parameters. To achieve radiolucency of bone, consider decalcification before scanning as follows in the next step. Troubleshooting 7 and 8.

17. Decalcification for radiolucent bones.
  a. Separate the head from the body.
  b. Prepare a 10% HCl solution or similar strong acid.
    ***Note:*** Weak acids may be used to perform decalcification, but sample treatment time will vary to achieve the optimal radiolucency.
  c. Transfer skinned sample to a 50 mL conical tube.
  d. Submerge the sample in acid solution.
  e. Incubate for 3 days at 20°C–22°C.with agitation.
  f. Remove the head from the acid solution and rinse with deionized water.
  g. Return the head to 1× PBS.
  h. Repeat steps 6 and 7.
18. Phosphotungstic acid (PTA) diffusion for soft tissue visualization.^7^^,^^8^
  a. Remove the skin from the head of the mouse.
  b. Submerge the sample into 1:40 (vol/vol) PTA:70% ethanol solution for 5–7 days based on sample size. Exchange PTA solution every 2–3 days.
    ***Optional:*** Graduated ethanol dehydration, while not required, may be performed prior to submerging the sample into PTA. If desired, the sample can sequentially be submerged over 12 h in 30%, 50%, and 70% ethanol.^7^ PTA is amphipathic, which means that it is soluble in both organic and aqueous solvents. While ethanol dehydration may introduce tissue shrinkage artifact, which is accounted for in the deformable registration in the Neurosimplicity Imaging Suite, PTA is less soluble in aqueous solutions than organic solutions. Therefore, we recommend immersion in PTA that has been dissolved in ethanol to improve PTA diffusion.^9^ Ethanol dehydration should be performed gradually to prevent tissue lysis.
  c. To evaluate if PTA has penetrated the center of the region of interest completely, perform a quick, low-resolution scout scan.
    i. If the scout scan displays incomplete penetration of PTA, which would be represented by absent attenuation in the center of the sample, for example the brain, return the sample to PTA/ethanol solution for an additional 1–2 days, then repeat the low-resolution scan. Otherwise, perform the high-resolution scan as described in step 6.
      ***Note:*** New scan parameters will need to be optimized for the PTA sample. Scan power will need to be increased compared to previous scans, and a denser filter should be applied.

### Visualization

**Timing: 12–24 h**

This section provides stepwise instructions for processing of acquired image data utilizing the Neurosimplicity Imaging Suite software.

19. Process acquired imaging data with the Neurosimplicity Imaging Suite for automatic deformable registration, feature extraction, and visualization. Troubleshooting 9 and 10.

***Note:*** If any of the iterative processing steps were skipped or not completed and thus those scans were not acquired, the imaging data acquired can still be processed by the Neurosimplicity Imaging Suite for extraction of the relevant features, i.e., visualization of vessels, brain, or bone.

## Expected outcomes

**Figure 5:**
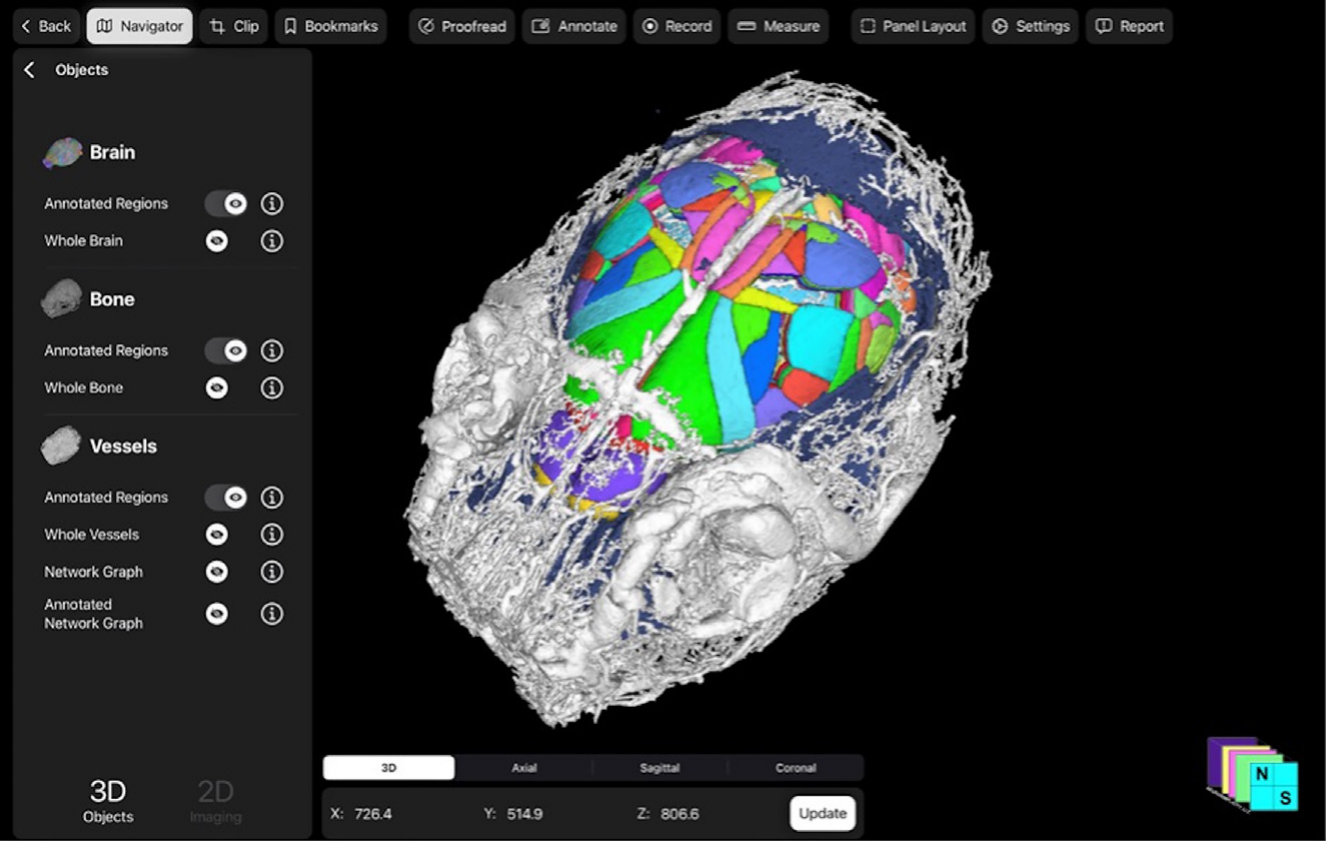
Final interactive 3D render of murine bone, brain, and vessels after iterative sample processing and imaging using the Neurosimplicity Imaging Suite Example coronal oblique view of the features, including bone (blue), brain registered to the ARA CCF v3 (multicolor), and vessels (white), were automatically extracted from each corresponding input dataset registered to the same space for visualization within the Neurosimplicity Imaging Suite. The calvarium and diploic veins have been toggled off to reveal the brain. An example of the initial user interface, which allows for toggling between 2D and 3D data views and displayed rendered objects through the navigator panel is shown. The interface supports touch and mouse interaction.

Upon completion of iterative sample processing and imaging as above, data processing using the Neurosimplicity Imaging Suite will automatically generate an interactive 3D render of the desired features to be analyzed. These features, including the brain, bone, and vessels, are automatically extracted by the software, and may be automatically registered to a selected atlas for labeling of identified objects or regions, such as the ARA CCF v3 for the murine brain (Figure 5).

**Figure 6:**
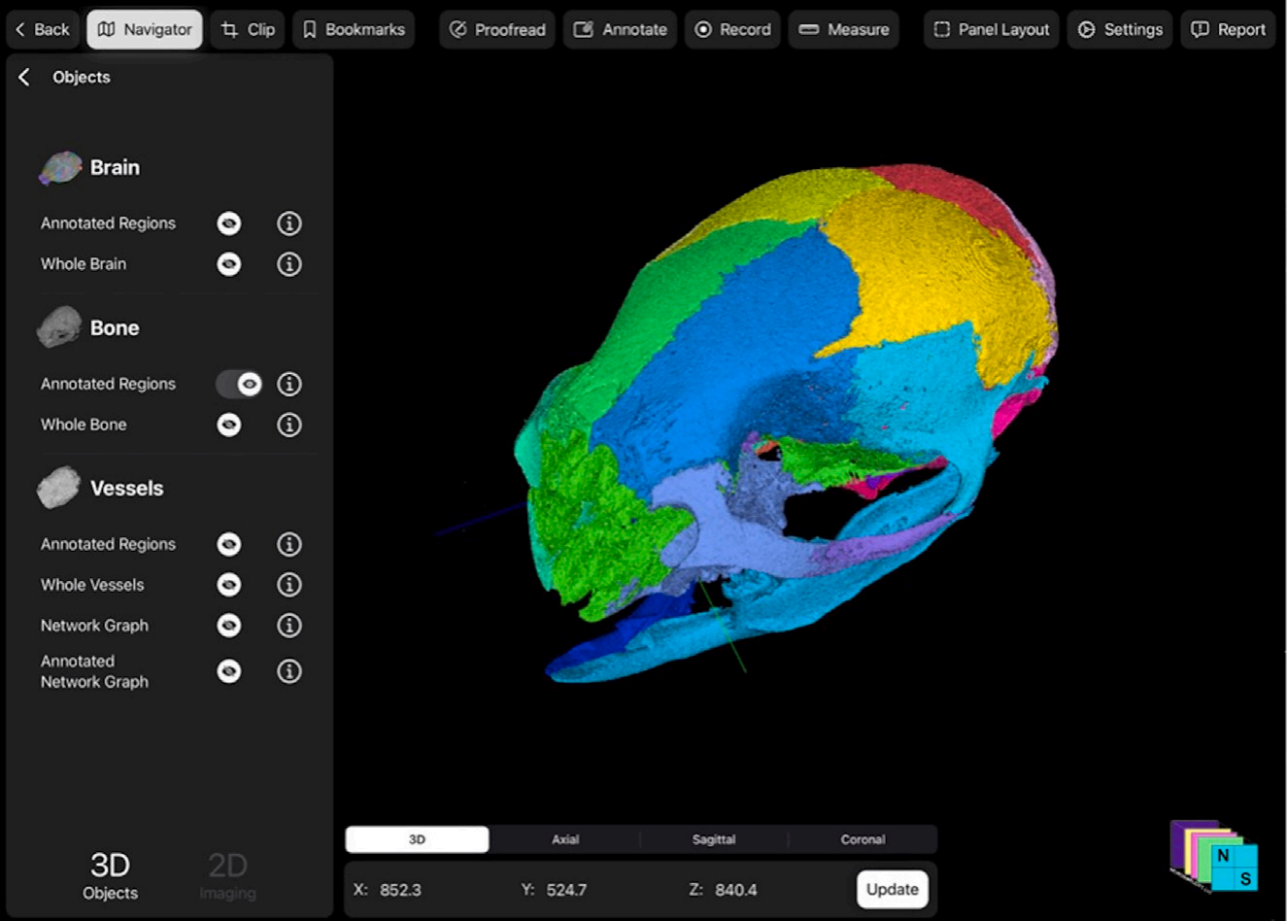
Example of selection of features or objects to be visualized and interacted with by toggling off brain and vessels to visualize the murine bone in detail Coronal oblique view of the automatically extracted and rendered skull (multicolor). Each bone comprising the skull is a separate object that can be toggled on and off and colored and interacted with accordingly.

Each feature may selectively be toggled on or off for further visualization through the navigator panel. Further, the user can interact with each object independently. When selecting for a specific anatomic object, such as bone, the 3D render remains interactive and can be viewed in several different orientations as seen on the bottom panel (Figure 6).

**Figure 7:**
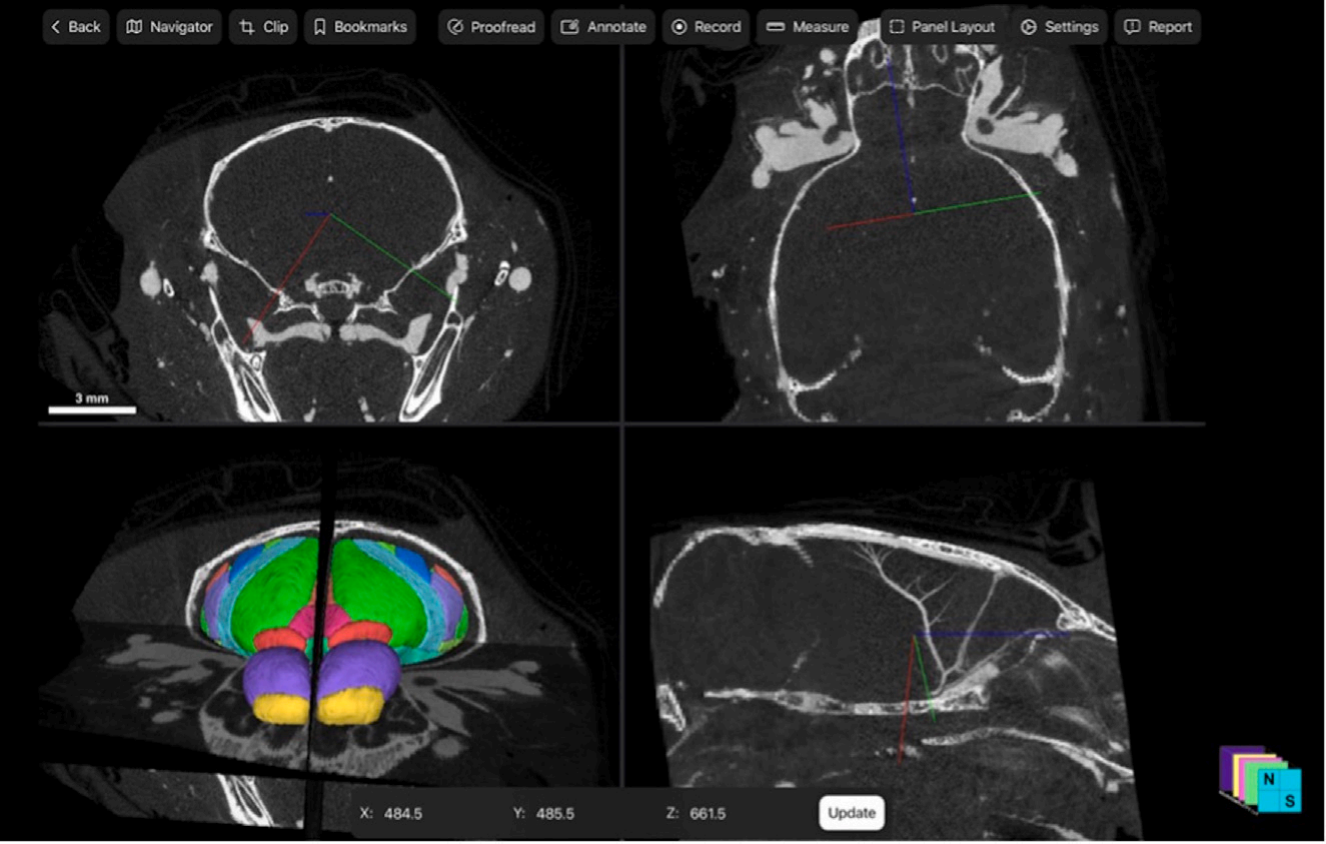
Four-panel view of 2D and 3D data acquired before decalcification The four-panel view of the Neurosimplicity Imaging Suite, which allows viewing and interaction with both 3D visualization and 2D raw data acquired before decalcification in orthogonal planes is shown. The following orientations are included: coronal (top left); axial (top right); and sagittal (bottom right). The 3D visualization of the extracted brain registered to the ARA CCF v3 is shown with corresponding 2D slices (bottom left). The corresponding location between 2D and 3D images is demonstrated in each panel with the multicolor crosshairs.

**Figure 8:**
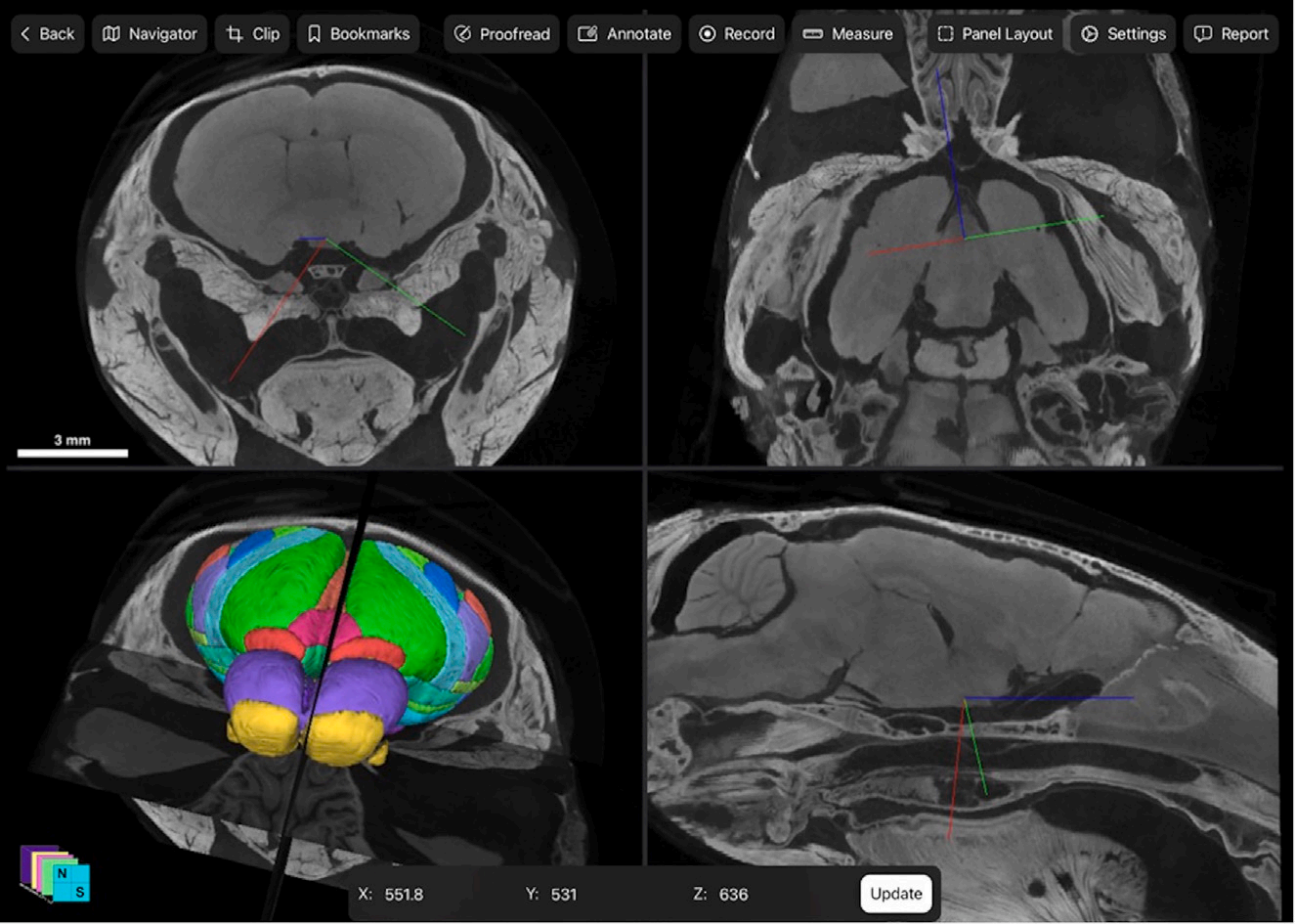
Four-panel view of 2D and 3D data acquired after PTA diffusion The four-panel view of the Neurosimplicity Imaging Suite, which allows viewing and interaction with both 3D visualization and 2D raw data acquired after decalcification and immersion in phosphotungstic acid (PTA) in orthogonal planes is shown. The following orientations are included: coronal (top left); axial (top right); and sagittal (bottom right). The 3D visualization of the extracted brain registered to the ARA CCF v3 is shown with corresponding 2D slices (bottom left). The corresponding location between 2D and 3D images is demonstrated in each panel with the multicolor crosshairs.

To visualize all the automatically extracted and rendered 3D objects in context of the 2D input data on the same screen, a four-panel view can be selected under “Panel Layout”. This view includes all toggled features side-by-side in orthogonal planes (Figures 7 and 8**).**

## Limitations

The method presented allows a nondestructive visualization of the *in situ* vasculature and surrounding anatomy of any organ in states of health or disease by combining terminal polymer casting of vessels with iterative sample processing, and image acquisition. While this workflow provides detailed appreciation of vascular anatomy, such as neurovascular interfaces, it ultimately requires animal sacrifice prior to imaging. Therefore, this method as presented may not be optimal for evaluating the dynamics of blood flow. *In vivo* imaging methods may be better for this purpose. Recent studies have used ultrasound (US) or contrasted or time-of-flight MRI methods for such purposes like velocity-based measurements of directionality of blood flow which help determine vessel type.^11^^,^^12^^,^^13^ While our *ex vivo* imaging method does not allow for assessment of blood flow, it does provide a uniform effect on vessel diameter through the use of sodium nitroprusside alone. The provision of anesthetics for *in vivo* methods, in comparison, may have complicated, unpredictable combinatorial effects on vasoconstriction and vasodilation.^14^^,^^15^ Thus, when performing manual or automated measurements of vessel caliber, we prefer and recommend uniform maximal vasodilation such as achieved herein with sodium nitroprusside. In animal models where this step may no have the desired effects, other agents may be used.^16^^,^^17^^,^^18^ Ultimately both *in vivo* and *ex vivo* methods have limitations on quantitative evaluation of vasculature like vessel diameter measurement. Due to significant discrepancies in image resolution, images obtained from our *ex vivo* method cannot be directly compared to *in vivo* imaging to compare changes in such metrics, yet the anatomy can still be compared. Therefore, our workflow is particularly suitable for morphological, structural, and developmental studies of vasculature and surrounding anatomy and can be applied to any organ or tissue type of interest. Finally, we chose the Neurosimplicity Imaging Suite to perform automatic deformable registration, feature extraction, and visualization on iteratively acquired imaging data of the same iteratively processed sample. This software platform is commercially available and leverages available open-source tools.^19^^,^^20^ While a paid license is required to use this platform, it is possible to view and interact with the output of each step of the imaging protocol presented herein using the software available with the micro-CT system.

## Troubleshooting

### Problem 1

#### Procedural step: Perfusion

Problem 1. After administration of Nitroprusside and/or Microfil®, leaking is evident from the site of cannulation.

#### Possible reasons

• The depth of the inserted needle is too shallow and may terminate prematurely before the suture line.
• The sutures meant to secure the needle are loose.
• Iatrogenic damage to the aorta has occurred during steps involving suturing or cannulation.

### Potential solution

• Re-insert or deepen the needle to a depth that reaches past the sutures.
• Re-secure the sutures by tightening those placed around the aorta without causing laceration.
• Select a position superior to the current site of cannulation and recannulate following steps 1d–1l.

### Problem 2

#### Procedural step: Perfusion

Problem 2. Microfil is not leaving the IVC as expected and is instead leaking from adjacent vessels.

#### Possible reasons

• Iatrogenic injury to the internal mamillary arteries may have occurred during the thoracotomy.
• Increased perfusion pressure during manual administration may have caused vessel rupture.
• Heparinization was not adequate.

### Potential solution

• Cannulate the segment of descending aorta within the abdominal cavity. Do not elevate the chest.
• Using a new sample, administer the Microfil slowly at a constant rate either manually or automatically with a syringe pump, if available. If the problem does not resolve, decrease the viscosity of the polymer.

### Problem 3

#### Procedural step: Perfusion

Problem 3. During Microfil administration, resistance within the syringe precludes meeting the perfusion endpoint.

#### Possible reasons

• The viscosity of the polymer mixture is not optimal.
• Blood clots formed in smaller vessels due to insufficient vascular flushing.
• Premature polymer curing occurred within any segment of the system, including the syringe, catheter tubing, or proximal vessels thereby impeding further perfusion. Since Microfil cures within 30 min of being mixed, perfusion should be initiated prior to this time point.

### Potential solution

• Prepare and perfuse a new sample with an optimal ratio of polymer mixture. Add more diluent to the mixture if needed.
• Prepare a new sample. After animal sacrifice, immediately flush the vessels with nitroprusside.
• If a new sample cannot be utilized, remove the existing catheter, recannulate the sample with a new syringe, catheter, and tubing system, and prepare a fresh Microfil solution for perfusion.

### Problem 4

#### Procedural step: Curing and fixation

Problem 4. Microfil appears to be uncured and floating within the fixative.

#### Possible reasons

• Polymer curing was not adequate. Longer times for curing and fixation may need to be considered if the viscosity of the solution was altered.

### Potential solution

• Allow the Microfil solution to cure for 120 min total by curing for an additional 60 min. Repeat the protocol with a new sample if Microfil extravasation occurred during curing.
• Invert the curing agent multiple times to promote adequate mixing prior to its addition to the solution.

### Problem 5

#### Procedural step: Curing and fixation

Problem 5. Sample freezing occurred during polymer curing.

#### Possible reasons

• Excess dry ice was utilized or the barrier between the sample and dry ice was absent.

### Potential solution

• Avoid placing the samples directly on dry ice. Alternatively, decrease the amount of dry ice used or use wet ice instead.

### Problem 6

#### Procedural step: Iterative processing and imaging

Problem 6. Tissue appears digested after HCl decalcification.

#### Possible reasons

• The HCl solution was too concentrated
• The sample was left in the HCl solution too long.

### Potential solution

• Discard the sample and start the process over with a lower concentration of HCl solution or a shorter incubation time at the decalcification step.
• Discard the sample and start the process over with EDTA calcification, which may require longer incubation.

### Problem 7

#### Procedural step: Iterative processing and imaging

Problem 7. Incomplete PTA penetration of the sample.

#### Possible reasons

• PTA diffusion is not complete.
• All available PTA in solution has been consumed.
• PTA solution has settled at the bottom of the tube.

### Potential solution

• Return the sample to PTA immersion.
• Exchange the PTA solution.
• Set the tube with the sample in solution on a rocker.

### Problem 8

#### Procedural step: Iterative processing and imaging

Problem 8. The acquired images appear blurry.

#### Possible reasons

• The sample shifted during the scanning process.
• The thawing of a cold sample may have precipitated movement during the scanning process.
• Sample dehydration, represented by loss of fluid in soft tissues, may have facilitated sample shifting during a prolonged scanning process.

### Potential solution

• Assess for gaps in the sample tube and use radiolucent materials such as Styrofoam to remove these gaps and further fasten the sample.
• Ensure the sample thaws to 20°C–22°C.and repeat the scans of the sample.
• Circumferentially cover the sample in parafilm to prevent fluid loss. If able, optimize the parameters to decrease scanning times.

### Problem 9

#### Procedural step: Iterative processing and imaging

Problem 9. Microfil appears indistinguishable from the bone.

#### Possible reasons

• Parameters for Micro-CT did not follow suggested settings.
• 3D reconstruction parameters were not optimized.
• Scans were output in 8-bit.
• Decalcification of the sample was not done.
• Air bubbles compromised the vascular system.

### Potential solution

• Assess and re-optimize scan parameters, to include the X-ray power and filter type/thickness.
• Re-adjust the dynamic range by reassessing the densest signal and setting the upper limit. The lower limit does not usually require re-adjustment as it is typically set to zero.
• To potentially improve segmentation of the polymer density by removing soft tissue and optimizing saturation of boney signal, adjust the dynamic range as follows: increase the lower limit and decrease the lower limit.
• Output the data in 16-bit format.
• Perform decalcification of the sample in a weak acid as suggested in the above steps for decalcification.

### Problem 10

#### Procedural step: Iterative processing and imaging

Problem 10. The Microfil signal appears discontinuous on Micro-CT, particularly in vessels of small caliber.

#### Possible reasons

• Air bubbles compromised the vascular system during perfusion.
• The transfer function has not been optimized.
• Blood clots formed in small vessels due to inadequate vessel flushing.
• Premature polymer curing occurred within any segment of the system impeded completed perfusion of the sample. Since Microfil cures within 30 min of being mixed, perfusion should be initiated prior to this time point.
• An inadequate volume of Microfil was administered into the sample.

### Potential solution

• Using a new sample, carefully avoid the introduction of air bubbles into the syringe, tubing, and catheter during administration of all relevant solutions.
• Alter the transfer function to optimize a threshold range in line with visualization of smaller vessels. Note: In order to visualize smaller vessels, the background noise may undergo a compensatory increase.
• Using a new sample, ensure that the nitroprusside is flushed completely until it is no longer seen exiting the IVC.
• Using a new sample, perfuse relevant solutions slowly and constantly either manually or automatically with a syringe pump, if available.
• The precise volume of Microfil required for optimal perfusion ultimately varies between samples, therefore, continue polymer injection until it appears exiting from the IVC.

### Problem 11

#### Procedural step: Visualization

Problem 11. The calvarium in the posterior region of the head has fractured, and a hematoma has developed.

#### Possible reasons

• Iatrogenic damage to the cranium occurred during animal sacrifice, with a greater likelihood during methods which cause physical damage to anatomy like cervical dislocation.

### Potential solution

• Perform animal sacrifice with carbon dioxide narcosis as described here with subsequent thoracotomy/laparotomy.

### Problem 12

#### Procedural step: Visualization

Problem 12. Only arteries or vessels of large caliber are evident on the acquired images.

#### Possible reasons

• The viscosity of the Microfil mixture was too thick.

### Potential solution

• Check the ratio of Microfil to diluent to polymerizing agent for a ratio that optimizes transport across capillaries.

## Resource availability

### Lead contact

Further information and requests for resources and reagents should be directed to and will be fulfilled by the lead contact, Jared S. Rosenblum at jared@neurosimplicity.com.

### Materials availability

This study did not generate new unique reagents.

## Data Availability

This paper does not report Standardized data types. All data reported in this paper will be shared by the lead contact upon request. This paper does not report original code. Any additional information required to reanalyze the data reported in this paper is available from the lead contact upon request.
